# High blood pressure as a risk factor for incident stroke among very old people: a population-based cohort study

**DOI:** 10.1097/HJH.0000000000001048

**Published:** 2016-09-01

**Authors:** Carl Hörnsten, Bodil Weidung, Håkan Littbrand, Bo Carlberg, Peter Nordström, Hugo Lövheim, Yngve Gustafson

**Affiliations:** a Department of Community Medicine and Rehabilitation, Geriatric Medicine; b Department of Public Health and Clinical Medicine, Medicine, Umeå University, Umeå, Sweden

**Keywords:** 80 and over, aged, atrial fibrillation, blood pressure, cerebrovascular disorders, risk factors, stroke, very old

## Abstract

**Introduction:**

High blood pressure (BP) increases the risk of stroke, but there is limited evidence from studies including very old people. The aim was to investigate risk factors for incident stroke among very old people.

**Methods:**

A prospective population-based cohort study was performed among participants aged at least 85 years in northern Sweden. The 955 participants were tested at their homes. BP was measured manually after 5-min supine rest. Incident stroke data were collected from medical charts guided by hospital registry, death records, and 5-year reassessments. Cox proportional hazards models were used.

**Results:**

The stroke incidence was 33.8/1000 person-years (94 stroke events) during a mean follow-up period of 2.9 years. In a comprehensive multivariate model, atrial fibrillation [hazard ratio 1.85, 95% confidence interval (CI) 1.07–3.19] and higher SBP (hazard ratio 1.19, 95% CI 1.08–1.30 per 10-mmHg increase) were associated with incident stroke overall. However, higher SBP was not associated with incident stroke in participants with SBP less than 140 mmHg (hazard ratio 0.90, 95% CI 0.53–1.53 per 10-mmHg increase). In additional multivariate models, DBP at least 90 mmHg (hazard ratio 2.45, 95% CI 1.47–4.08) and SBP at least 160 mmHg (vs. <140 mmHg; hazard ratio 2.80, 95% CI 1.53–5.14) were associated with incident stroke. The association between BP and incident stroke was not affected by interactions related to sex, dependence in activities of daily living, or cognitive impairment.

**Conclusion:**

High SBP (≥160 mmHg) and DBP (≥90 mmHg) and atrial fibrillation appeared to be risk factors for incident stroke among very old people.

## INTRODUCTION

The prevalence and incidence of stroke are markedly high among very old people (those ≥80 years) [1,2]. Stroke care for very old patients will likely become more important because of the projected expansion of the age group [3].

Hypertension did not appear to be a risk factor for incident stroke among very old people in three population-based cohort studies [4–6], although another cohort study found that higher SBP (analyzed as a continuous measure) was associated with incident stroke [7]. Furthermore, the association between hypertension and stroke mortality appeared to be notably weaker among very old people compared with younger age groups in a large meta-analysis [8].

Risk factors for stroke have been investigated widely in younger populations [9], but few studies have investigated the very old age group. Meanwhile, risk factors for stroke in younger populations cannot reasonably be assumed to apply to the very old, considering the physiological, pathological, and social changes associated with aging. To our knowledge, the present study is one of the largest population-based cohort studies to date investigating high blood pressure (BP), together with multiple other relevant factors, as risk factors for incident stroke among very old people.

## METHODS

### Setting and procedure

The population-based Gerontological Regional Database (GERDA) cohort study was initiated to investigate the health/disease status of very old people and factors leading to good aging in this population. Half of all 85-year-olds, all 90-year-olds, and all participants aged at least 95 years in selected municipalities of Västerbotten County, northern Sweden, were invited to participate. The first round of data collection was performed in an urban municipality in 2000 and in five adjacent rural municipalities in 2002. A second round of data collection was performed in 2005 and 2007 with the same inclusion criteria, meaning that all surviving past participants were eligible for reparticipation in older age groups. A third data collection was performed in 2010 and 2012.

Eligible individuals were informed about the study by postal mail and then contacted by telephone. Participants underwent assessments and physical tests during visits in residences and institutional care facilities. Medical charts were reviewed, and relatives and institutional care staff were contacted for supporting information when needed. Oral consent to participate was acquired during telephone calls and home visits. When cognitive impairment was suspected, a relative was also asked to provide consent. Examiners were nurses, physical therapists, physicians, and medical students. The Regional Ethical Review Board of Umeå/Sweden approved the study (2014-221-31m).

### Participants

Five hundred and twenty-seven individuals were eligible for participation in 2000/2002, 612 were eligible in 2005/2007, and 796 were eligible in 2010/2012. When the same individual was eligible in multiple data collection rounds, they were included from their first home visitation. Of 1935 potential participants, 419 were removed due to being eligible in multiple rounds of data collection and 137 died before they could be contacted. Of 1379 eligible individuals, 160 declined to participate, 8 were excluded for other reasons, and 256 declined home visitation, resulting in a final sample of 955 individuals. This sample included 69% of eligible individuals who were alive when contacted. It contained slightly more men compared with the remaining eligible individuals (34.1 vs. 28.3%; chi-squared test, *P* = 0.028), but the age distribution was similar (*n* = 424; Welch test, *P* = 0.613).

### Follow-up

The follow-up period was up to 5 years. Registry information was collected from the government-run healthcare provider responsible for all inpatient care in the county. Causes of death were collected from the National Board of Health and Welfare. Records of inpatient care after baseline assessment with International Classification of Diseases (10th version; ICD-10) code prefixes I60, I61, I63, I64, and H34; causes of death registered with ICD-10 code prefixes I60, I61, I63, I64, H34, I67, and G45; and subsequent diagnoses of stroke or transient ischemic attack within the last 5 years among people who participated in multiple GERDA assessments were compiled for all participants.

A physician comprehensively reviewed the digital medical charts of individuals identified in this manner. All discrete stroke events described in medical charts were recorded. Of the 94 individuals with one or more incident strokes, 71 were identified using registry information (29 uniquely), 50 by cause of death (19 uniquely), and 20 by repeat study assessment (three uniquely).

### Definition of stroke

Individuals with current stroke diagnoses in their medical charts after inclusion into the study were considered to have experienced incident stroke. A current stroke diagnosis was understood as discrete onset of neurological deficit that was designated as a stroke, but not a transient ischemic attack, by a treating physician. Stroke events were classified as ischemic, hemorrhagic, or unclassified. Events judged to be traumatic intracerebral hemorrhage were not considered to be strokes.

A previous stroke was considered to be present if the medical charts included a stroke diagnosis or if the individual, a caregiver, or a relative reported the diagnosis and it was found to be credible based on supporting information from medical charts or other assessments.

### Physical measurements

SBP and DBP were measured manually with a calibrated sphygmomanometer after 5-min supine rest. The left arm was used to measure BP. BP was measured at one point in time, with no temporally separated follow-up measurements. Pulse pressure (PP) was calculated as SBP − DBP. Mean arterial pressure (MAP) was calculated as SBP/3 + DBP × 2/3.

Weight was measured with a calibrated digital scale. Length was measured with a measuring stick.

Usual gait speed was measured over 2.4 m. Participants were instructed to walk at usual pace and permitted to use mobility aids when necessary. A mean of two tests was calculated. Individuals who were unable to perform the test due to physical impairment were assigned an imputed value of 0.01 m/s.

The chair stand test consisted of the individual being asked to rise up and sit down three times without support from an initial sitting position.

### Assessments

The 15-item Geriatric Depression Scale (GDS-15) [10] was used to screen for depressive symptoms. Scores at least 5 are considered to indicate depression.

The mini–mental state examination (MMSE) [11] was used to test for cognitive impairment. Scores range from 0 to 30, with higher scores indicating better cognitive function.

The Barthel Activities of Daily Living (B-ADL) index [12] was used to assess dependence in ADL. The scale ranges from 0 to 20, with a score of 20 indicating independence in personal ADL.

The Mini–Nutritional Assessment (MNA) [13] was used to assess nutrition. Scores range from 0 to 30, with scores less than 17 indicating malnutrition and at least 24 indicating normal nutrition.

### Other diagnoses/definitions

All other diagnoses, including atrial fibrillation, congestive heart failure, diabetes, rheumatic disease, dementia, and delirium (last month), were based on information from assessments conducted during home visits and records from hospitals, general practitioners, and institutional care facilities. A specialist in geriatric medicine evaluated diagnoses. Current medications were asked about during the home visit and confirmed in medical records. Smoking status was investigated with the question ‘Do you smoke?’, answered with ‘yes’, ‘no’, or ‘I have smoked previously’.

### Statistical analyses

R 3.0.2 (The R Foundation, Vienna, Austria) was used for statistical analyses. Associations with incident stroke were analyzed with Cox proportional hazards models. Observation time was calculated as the interval from study inclusion to stroke event, death, or end of the follow-up period (≤5 years). The assumption of proportional hazards was tested by regression of Schoenfeld residuals against time. For the outcome of incident stroke, univariate models for the variables of age (continuous measure; *P* = 0.046), DBP (continuous measure; *P* = 0.028), B-ADL index less than 20 (*P* = 0.040), rheumatic disease (*P* = 0.047), and diuretic use (*P* = 0.027) showed signs of time dependence.

Baseline variables thought to be associated with incident stroke based on previous research [9,14] and clinical experience were tested using univariate models. These variables included traditional stroke risk factors such as atrial fibrillation, diabetes, smoking status, BMI, BP levels, and gait speed as an indirect measure of physical activity level, but also nontraditional risk factors such as depression and rheumatic disease. Factors that are generally influential in geriatric populations that were thought to be relevant, such as cognitive impairment, ADL dependency, nutritional status, and ongoing medical treatment, were also tested.

All continuous variables were tested both alone and with corresponding quadratic terms to examine possible nonlinear associations. Continuous variables were also tested as categorical variables according to traditional cutoffs. For SBP, categories corresponding to ‘Stage 1 hypertension’ (140–159 mmHg), ‘Stage 2 hypertension’ (≥160 mmHg), and a reference range (<140 mmHg) were used, resulting in three equally sized groups. For DBP, categories corresponding to ‘Stage 1 hypertension’ or ‘Stage 2 hypertension’ (≥90 mmHg) and a reference range (<90 mmHg) were used. The groups for DBP were combined to facilitate statistical testing, considering the limited number of participants with DBP at least 90 mmHg.

Variables associated significantly with incident stroke (Table 1) were entered into multivariate models. No multivariate model included time-dependent terms. Basic, intermediate, and comprehensive models were used, with the progressive introduction of variables and dropouts due to missing values. Dementia and delirium (in the last month) were not included in the main multivariate models due to strong correlations (≥0.5) with MMSE. Quadratic B-ADL index was not included due to strong correlations with MNA and MMSE scores and gait speed. SBP, DBP, PP, and MAP were included in separate multivariate models.

Models of different BP measures, with and without interaction terms, were used to analyze subgroup differences according to sex (male vs. female), ADL dependence (B-ADL index <20 vs. 20), and cognitive impairment (MMSE score <24 vs. ≥24). The interaction models took the form of ‘previous stroke + atrial fibrillation + heart failure + BP + “subgroup variable” + BP × “subgroup variable” (interaction term)’. The interaction models were compared with models without interaction terms using likelihood ratio tests. Continuous measures of BP were used, as recommended previously [15]. *P* less than 0.05 was considered to indicate significance.

## RESULTS

**FIGURE 1:**
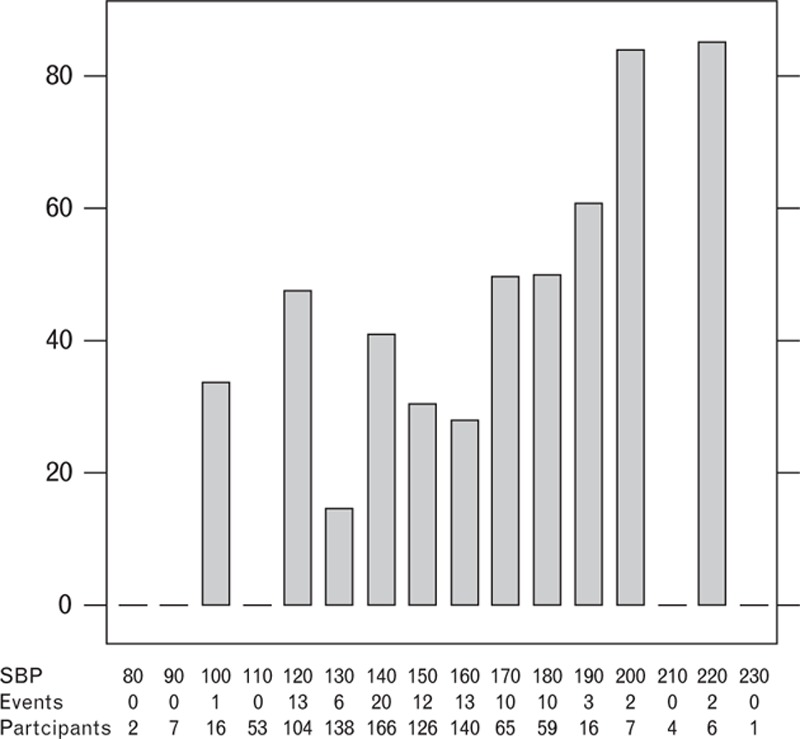
Stroke events per 1000 person-years for 10-mmHg SBP intervals, from 80 to 240 mmHg. The intervals are left-closed and right-open. Starting values and numbers of events and participants are presented for each interval.

Baseline characteristics are presented in Table 1. At baseline, 219/955 (22.9%) people had had previous strokes. During a mean follow-up period of 2.9 years, 94 (9.8%) people had incident strokes [70 (74.5%) ischemic, 4 (4.2%) hemorrhagic, and 20 (21.3%) unclassified]. Of those affected, 63 (67.0%) were admitted to hospitals, 10 (10.6%) to small rural hospitals, and 21 (22.4%) were not admitted to hospitals. Emergency computed tomography examinations were performed in 66 (70.2%) of these cases. Thirty-six of 94 (38.3%) individuals who suffered strokes died within 30 days. Per 1000 person-years, 52.9 strokes occurred in people with and 28.5 strokes occurred in those without previous stroke, resulting in 33.8 events overall (Table 2). Figure 1 presents the number of strokes per 1000 person-years for each 10-mmHg SBP interval.

In univariate models, incident stroke was associated with atrial fibrillation, higher SBP per mmHg-increase, DBP at least 90 mmHg, and higher MAP per mmHg-increase (Table 1). However, SBPs at least 160 mmHg (*P* = 0.051) and 140–159 mmHg compared with less than 140 mmHg and higher DBP per mmHg-increase were NS factors. Incident stroke was also associated with previous stroke, congestive heart failure, slower usual gait speed, lower MNA score, higher GDS-15 score, MMSE score less than 18 (vs. ≥24), dementia, and delirium (Table 1). In additional quadratic models, a nonlinear association between B-ADL index and incident stroke was observed (quadratic term <1, suggesting a concave association, reaching midinterval maximum). Incident stroke was not associated with the use of beta blockers, calcium blockers, AT1 blockers, ACE inhibitors, statins, acetylsalicylic acid, or warfarin (Table 1).

Atrial fibrillation and a higher SBP per mmHg-increase were associated consistently with incident stroke in a basic multivariate model adjusted for prevalent diseases, an intermediate model adjusted for prevalent diseases and MNA and MMSE scores, and a comprehensive model adjusted for prevalent diseases, MNA, MMSE, and GDS-15 scores and gait speed (Table 3). Higher SBP per mmHg-increase was also associated with incident stroke in a comprehensive model including only participants with SBP at least 140 mmHg [hazard ratio 1.17, 95% confidence interval (CI) 1.03–1.33/10-mmHg increase], although not among those with SBP less than 140 mmHg (hazard ratio 0.90, 95% CI 0.53–1.53/10-mmHg increase). In additional comprehensive multivariate models containing variables not included in the main models due to strong correlations, with variables showing correlations at least 0.5 removed, incident stroke was not associated with dementia, delirium, or quadratic B-ADL index (data not shown).

**FIGURE 2:**
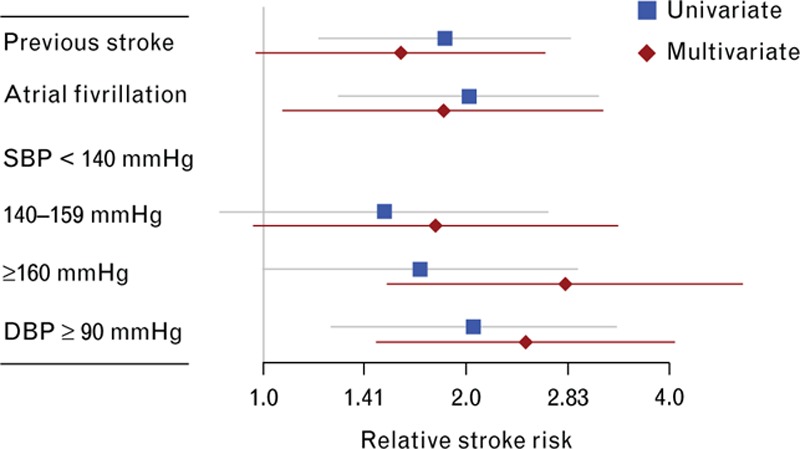
Forest plot comparing univariate and multivariate associations with incident stroke. A logarithmic scale was used. Cox proportional hazards models were used. Multivariate models included atrial fibrillation, previous stroke, congestive heart failure, categorical mini–mental state examination, Mini–Nutritional Assessment score, gait speed, and Geriatric Depression Scale score. The multivariate model of previous stroke and atrial fibrillation also included continuous SBP.

In multivariate analyses, employing SBP as a categorical variable, a consistent association was observed between SBP at least 160 mmHg (vs. <140 mmHg) and incident stroke; SBP of 140–159 mmHg was not a significant factor in these analyses (Table 4). Separate multivariate analyses including other BP measures revealed consistent associations between incident stroke and DBP at least 90 mmHg and higher DBP, PP, and MAP per mmHg-increase (Table 4). Univariate and multivariate associations with incident stroke are presented in Figure. 2.

The associations of incident stroke with SBP, DBP, PP, and MAP were not affected by interactions related to sex, ADL dependence, or cognitive impairment (all *P* > 0.25).

## DISCUSSION

In the present study, 22.9% of participants had had previous strokes at baseline, and the stroke incidence was 33.8/1000 person-years. High BP including measures of SBP, DBP, PP, and MAP, and atrial fibrillation were associated independently with incident stroke. The association with SBP was linear overall, but was NS for participants with SBPs less than 140 mmHg.

The stroke incidence among very old people in the present study was higher compared with the Framingham study [6] (18.0/1000 person-years, 1948–1982) and the Oxford Vascular Study [2] (18.2/1000 person-years, 1981–1984; 16.5/1000 person-years, 2000–2004), but lower compared with the H70 study [7] (57.2/1000 person-years, 1986–1989). The incidence observed in the present study was high, despite the decreasing stroke incidence in recent decades [2].

The finding of independent associations between incident stroke and SBP at least 160 mmHg (vs. <140 mmHg), DBP at least 90 mmHg, and higher SBP, DBP, PP, and MAP per mmHg-increase is not consistent with the results of most previous population-based cohort studies, which have found no association between hypertension and incident stroke among very old people [4–6]. These results, however, agree with those of a population-based cohort study [7] of 401 elderly individuals, which showed that SBP as a continuous measure was associated with incident stroke in analyses adjusted for sex and depression. In contrast to most observational studies, the randomized controlled Hypertension in the Very Elderly Trial (HYVET) showed that antihypertensive therapy was associated with decreased stroke mortality and borderline associated with a decreased stroke incidence [16]. A meta-analysis that included HYVET data subsequently found that antihypertensive therapy reduced stroke incidence [17].

Given the notable exclusion of people with cognitive and/or physical disability from the HYVET and other trials, and the mostly negative results from population-based cohort studies among very old people [4–6], whether hypertension truly increases stroke risk in representative populations of very old people remains unclear. Our population-based data, from a sample that included many people with disabilities, indicate that high BP is a risk factor for incident stroke among very old people. Furthermore, the association between BP and incident stroke appeared to be similar in people with and without ADL dependence or cognitive impairment.

Stroke risk appeared to increase linearly with SBP values overall, but this finding does not indicate a clinically meaningful difference between high and low BP in all intervals. For example, a linear increase in risk was observed for individuals with SBP at least 140 mmHg, but not for those with SBP less than 140 mmHg. From another perspective, the results of categorical models suggested that SBP at least 160 mmHg (vs. <140 mmHg) increased stroke risk, but the difference between SBPs of 140–159 and less than 140 mmHg had no clear effect on this risk. Thus, a target SBP less than 160 mmHg appears to be beneficial according to our observational data, but it is less clear if a target SBP less than 140 mmHg would further reduce the stroke risk among very old people.

In the present study, SBP at least 160 mmHg (vs. <140 mmHg) was not associated with incident stroke in the univariate model, but this association was strong in the comprehensive multivariate model. In addition, associations of incident stroke with SBP 140–159 mmHg (vs. <140 mmHg) and DBP at least 90 mmHg appeared to grow stronger in multivariate models. The difference between univariate and multivariate results suggests that confounding factors may obscure the association between high BP and incident stroke, resulting in higher hazard ratios in multivariate models. The negative results of some previous studies may be partly explained by modest adjustment for BP confounders.

The finding of an independent association between atrial fibrillation and incident stroke among very old people is in line with the results of a large population-based study [6]. In the present study, congestive heart failure, low MNA score, MMSE score less than 18, low gait speed, and high GDS-15 score were associated with incident stroke, but not independently of each other. Some of these factors may increase stroke risk, individually or collectively as a measure of geriatric multimorbidity, but we could not establish them as independent risk factors.

Antihypertensive patients and other drugs used for stroke prevention were not associated with incident stroke. Although it may seem logical to expect these treatments to be associated with a reduced stroke incidence, such associations are likely to be mitigated by worse cardiovascular risk profiles in the treated individuals.

### Limitations

Case ascertainment through medical chart review may have excluded some actual strokes that were not documented or included strokes that were mischaracterized. Diagnostic accuracy could theoretically have been improved with repeated clinical/radiological examinations, but this approach would have raised logistical and ethical issues in the very old age group. However, the investigation of medical charts was guided by registry information and complemented by reassessment of those who participated in 5-year follow-up data collection rounds, increasing sensitivity in the detection of incident stroke.

The present study investigated a comprehensive selection of possible risk factors for incident stroke, but data on some relevant risk factors were not available. As no blood testing was carried out, lipid levels, renal profile, and markers of hypertension-related organ damage could not be investigated as risk factors. As no baseline radiology was performed, the measure of previous stroke reflects only known clinical cases. These omissions are largely inherent to large population-based studies involving data collection from participants at their homes. In turn, home visitation for baseline assessments is likely key to the achievement of an acceptable participation rate of representative very old individuals.

BP was measured while participants were supine in the present study; in other observational studies, it has generally been measured while participants are seated [4–7]. This difference raises the possibility of a systematic difference in BP that may have affected our results compared with other studies.

The setting in northern Sweden included both urban and rural municipalities, encompassing individuals from a broad socioeconomic range. A large population-based study among participants aged less than 75 years found a higher concentration of cardiovascular disease in northern Sweden than in many other western European countries some decades ago, but this difference seems to have attenuated over the years, possibly due to improved medical treatment regionally [18]. The setting in the present study should be broadly comparable with other western European settings.

In conclusion, the prevalence and incidence of stroke were high in this representative sample of very old people. High SBP (≥160 mmHg) and DBP (≥90 mmHg) and atrial fibrillation appeared to be risk factors for incident stroke in this population. The association between BP and incident stroke appeared to be similar in men and women and in people with and without ADL dependence and cognitive impairment. Confounding factors may obscure the association between BP and incident stroke unless accounted for.

## ACKNOWLEDGEMENTS

Funding for the study was received from The Fund for Stroke Research in Norrland, Bothnia Atlantica Programme, European Regional Development Fund, Swedish Research Council (K2014-99X-22610-01-6), Swedish Dementia Association, and Swedish Stroke Association.

### Conflicts of interest

There are no conflicts of interest.

## Figures and Tables

**TABLE 1:** Baseline characteristics and univariate associations with incident stroke

	Baseline	Univariate models	
	*n* = 955 (%)	Hazard ratio (95% CI)	*P*
Age	89.3 ± 4.7	1.02 (0.97–1.07)	0.389
Women	629 (65.9)	1.05 (0.68–1.62)	0.838
Previous stroke	219 (22.9)	1.86 (1.21–2.85)	0.004
SBP (per 10-mmHg increase)	146.2 ± 23.0	1.10 (1.01–1.21)	0.028
SBP ≥ 160 mmHg (vs. <140 mmHg)	298 (32.7)	1.71 (1.00–2.92)	0.051
SBP 140–159 mmHg	292 (32.1)	1.51 (0.86–2.65)	0.147
DBP (per 10-mmHg increase)	74.2 ± 11.5	1.17 (0.97–1.40)	0.096
DBP ≥ 90 mmHg	119 (13.1)	2.05 (1.26–3.34)	0.004
PP (per 10-mmHg increase)	72.1 ± 19.6	1.09 (0.98–1.21)	0.119
MAP (per 10-mmHg increase)	98.2 ± 13.4	1.19 (1.02–1.39)	0.027
Barthel ADL index (score)	16.4 ± 5.5	0.99 (0.94–1.03)	0.508
Barthel ADL index <20	526 (55.7)	1.43 (0.95–2.15)	0.090
Barthel ADL index linear		1.33 (1.03–1.72)	0.030
Barthel ADL index quadratic		0.99 (0.98–1.00)	0.015
Usual gait speed (m/s)	0.5 ± 0.3	0.37 (0.16–0.83)	0.016
Chair stand, able to	567 (64.1)	0.74 (0.48–1.16)	0.190
BMI (kg/m^2^)	25.2 ± 4.4	0.97 (0.92–1.02)	0.224
BMI < 23 kg/m^2^ (vs. 23–29 kg/m^2^)	291 (32.1)	0.91 (0.57–1.46)	0.694
BMI ≥ 30 kg/m^2^	138 (15.2)	0.78 (0.43–1.44)	0.430
MNA (score)	23.6 ± 4.3	0.95 (0.91–1.00)	0.047
MNA < 17 (vs. ≥24)	75 (8.3)	1.35 (0.54–3.38)	0.527
MNA 17–23	300 (33.0)	1.35 (0.87–2.10)	0.178
GDS (score)	3.6 ± 2.5	1.10 (1.01–1.19)	0.024
GDS ≥ 5	232 (28.4)	1.44 (0.91–2.29)	0.117
Rheumatic disease	136 (14.2)	0.84 (0.45–1.57)	0.585
Atrial fibrillation	209 (21.9)	2.02 (1.29–3.15)	0.002
Diabetes	148 (15.5)	1.32 (0.78–2.24)	0.298
CHF	278 (29.2)	1.68 (1.09–2.58)	0.018
Current smoker	32 (3.4)	1.52 (0.62–3.75)	0.361
Ever-smoker	330 (35.0)	0.92 (0.59–1.41)	0.691
MMSE (score)	21.2 ± 7.6	0.98 (0.95–1.01)	0.138
MMSE <18 (vs. ≥24)	225 (24.3)	1.96 (1.16–3.34)	0.013
MMSE 18–23	248 (26.7)	1.28 (0.79–2.07)	0.309
Dementia	321 (33.6)	1.74 (1.13–2.69)	0.012
Education ≥8 years	221 (24.2)	1.28 (0.82–2.01)	0.276
Delirium (last month)	206 (21.6)	1.70 (1.01–2.87)	0.046
Neuroleptics	111 (11.6)	1.52 (0.85–2.74)	0.160
Acetyl salicyl acid	396 (41.5)	1.27 (0.85–1.90)	0.248
Warfarin	55 (5.8)	0.93 (0.38–2.28)	0.866
Diuretics	484 (50.7)	1.44 (0.96–2.17)	0.078
Benzodiazepines	232 (24.3)	1.12 (0.70–1.78)	0.635
Opioids	143 (15.0)	1.27 (0.73–2.20)	0.402
Steroids	169 (17.7)	0.79 (0.43–1.45)	0.451
Antidepressants	171 (17.9)	1.14 (0.67–1.96)	0.627
Statins	87 (9.1)	0.69 (0.30–1.57)	0.373
Beta blockers	361 (37.8)	1.17 (0.77–1.76)	0.459
Calcium blockers	142 (14.9)	0.94 (0.53–1.66)	0.830
ACE inhibitors	177 (18.5)	1.30 (0.77–2.17)	0.325
AT1 blockers	70 (7.3)	0.66 (0.27–1.63)	0.367

Count denominators may change because of missing values. Univariate Cox proportional hazards models were used. ACE, angiotensin-converting enzyme; ADL, activities of daily living; CHF, congestive heart failure; CI, confidence interval; GDS, Geriatric Depression Scale; MAP, mean arterial pressure; MMSE, mini–mental state examination; MNA, Mini–Nutritional Assessment; PP, pulse pressure.

**TABLE 2:** Stroke events per 1000 person-years

	Strokes	Observed years	Strokes per 1000 person-years
Age 85	44	1448.5	30.4
Age 90	33	862.8	38.2
Age ≥95 years	17	468.2	36.3
Women	65	1890.2	34.4
Men	29	889.3	32.6
Previous stroke	32	605.3	52.9
Stroke free	62	2174.2	28.5
SBP ≥ 160 mmHg	40	974.9	41.0
SBP 140–159 mmHg	32	881.0	36.3
SBP < 140 mmHg	20	842.8	23.7
DBP ≥ 90 mmHg	21	341.1	61.6
DBP < 90 mmHg	71	2349.4	30.2
Atrial fibrillation	28	495.8	56.5
No atrial fibrillation	66	2283.7	28.9
CHF	32	666.2	48.0
No CHF	62	2111.9	29.4
MMSE < 18	21	411.4	51.0
MMSE 18–23	27	748.1	36.1
MMSE ≥ 24	45	1573.4	28.6

CHF, congestive heart failure; MMSE, mini–mental state examination.

**TABLE 3:** Multivariate associations with incident stroke

	Basic model, *n* = 909 (92 events)		Intermediate model, *n* = 869 (89 events)		Comprehensive model, *n* = 759 (82 events)	
	Hazard ratio (95% CI)	*P*	Hazard ratio (95% CI)	*P*	Hazard ratio (95% CI)	*P*
SBP (per 10-mmHg increase)	1.16 (1.06–1.27)	0.001	1.19 (1.09–1.31)	<0.001	1.19 (1.08–1.30)	<0.001
Atrial fibrillation	1.78 (1.09–2.93)	0.022	1.79 (1.08–2.99)	0.025	1.85 (1.07–3.19)	0.027
Previous stroke	1.77 (1.13–2.77)	0.013	1.61 (1.01–2.56)	0.043	1.60 (0.98–2.63)	0.062
Congestive heart failure	1.50 (0.94–2.41)	0.092	1.56 (0.96–2.52)	0.071	1.37 (0.82–2.30)	0.227
MMSE < 18 (vs. ≥24)			1.82 (0.95–3.50)	0.073	1.50 (0.72–3.12)	0.275
MMSE 18–23			1.42 (0.86–2.33)	0.170	1.36 (0.81–2.29)	0.240
MNA (score)			0.97 (0.92–1.03)	0.405	0.97 (0.91–1.04)	0.434
Gait speed (m/s)					0.39 (0.13–1.16)	0.092
GDS					1.04 (0.95–1.14)	0.394

Multivariate Cox proportional hazards models were used. CI, confidence interval; GDS, Geriatric Depression Scale; MMSE, mini–mental state examination; MNA, Mini-Nutritional Assessment.

**TABLE 4:** Multivariate associations with incident stroke for additional blood pressure measures

	Basic models		Intermediate models		Comprehensive models	
	Hazard ratio (95% CI)	*P*	Hazard ratio (95% CI)	*P*	Hazard ratio (95% CI)	*P*
SBP ≥ 160 mmHg (vs. <140 mmHg)	2.20 (1.27–3.84)	0.005	2.51 (1.43–4.42)	0.001	2.80 (1.53–5.14)	<0.001
SBP 140–159 mmHg	1.63 (0.93–2.86)	0.089	1.60 (0.90–2.85)	0.109	1.80 (0.97–3.34)	0.062
DBP ≥ 90 mmHg	2.08 (1.28–3.41)	0.003	2.33 (1.41–3.85)	<0.001	2.45 (1.47–4.08)	<0.001
DBP (per 10-mmHg increase)	1.21 (1.01–1.45)	0.037	1.24 (1.04–1.49)	0.018	1.26 (1.05–1.52)	0.013
PP (per 10-mmHg increase)	1.14 (1.03–1.27)	0.015	1.18 (1.05–1.31)	0.004	1.17 (1.04–1.31)	0.006
MAP (per 10-mmHg increase)	1.27 (1.09–1.48)	0.002	1.32 (1.13–1.54)	<0.001	1.32 (1.13–1.54)	<0.001

Multivariate Cox proportional hazards models were used. Basic models included atrial fibrillation, previous stroke, and congestive heart failure. Intermediate models additionally included categorical mini–mental state examination, and Mini-Nutritional Assessment score. Comprehensive models additionally included Geriatric Depression Scale score and usual gait speed. CI, confidence interval; MAP, mean arterial pressure; PP, pulse pressure.

## Reviewers’ Summary Evaluations

### Reviewer 1

This prospective population-based cohort study has investigated the potential role of various risk factors for stroke incidence in very old subjects, aged ≥85 years old, who were living in Northern Sweden. The analysis confirmed that systolic blood pressure ≥160 mmHg increased the risk of stroke compared to those individuals with systolic blood pressure <140 mmHg, however the difference between other categories of systolic blood pressure (e.g. 140–159 and <140 mmHg) had no clear effect on this risk. This adds another relevant piece of evidence about the blood pressure targets to be achieved to reduce stroke risk in very old people.

### Reviewer 2

The authors address a somewhat neglected though clinically and therapeutically important question: the influence of high (>160 mmHg) vs lower (>140 mmHg) blood pressure on incident stroke in a very old (<85 years) population. In uni- and multivariate analyses, they show a relationship of stroke with the increase in blood pressure, particularly, a significant increase of stroke incidence at BP levels above 160 mmHg vs levels below 140 mmHg. In this analysis, diastolic BP above 90 mmHg also adds to increased stroke incidence.

The manuscript adds a significant piece of knowledge to the management of very old patients with increased BP levels. Some methodological shortcomings inherent to such a study, e.g. lack of lipid measurements, renal (dys-) function analysis or markers of organ damage, have to be accepted in view of the limitations in data acquisition. Somewhat intriguing is the lack of an association with incident stroke in the systolic range of 140-160 mmHg.
